# Serotyping of *Streptococcus pneumoniae* isolated from Tehran by Multiplex PCR: Are serotypes of clinical and carrier isolates identical?

**Published:** 2013-09

**Authors:** Seyed Fazlollah Mousavi, Saman Nobari, Fatemeh Rahmati Ghezelgeh, Hamid Lyriai, Pantea Jalali, Fereshteh Shahcheraghi, Mahvash Oskoui

**Affiliations:** Department of Bacteriology & Microbiology Research Center, Pasteur Institute of Iran, Tehran, Iran

**Keywords:** *Streptococcus pneumoniae*, Multiplex PCR, Serotyping

## Abstract

**Background and Objectives:**

*Streptococcus pneumoniae* is the most common cause of invasive infections among both young children and elderly people. Common serotypes causing invasive diseases and the emergence of carriers of *Streptococcus pneumoniae* in Iran is not yet known. Past-vaccine surveillance studies of serotype prevalence patterns in Iran are necessary to monitor the epidemiology of *Streptococcus pneumoniae*. Because of variation of pneumococcal serotypes in different geographical regions, in this study we evaluated common serotypes causing pneumococcal infections and healthy carrier children in Tehran by Multiplex PCR.

**Materials and Methods:**

A total of 150 nasopharyngeal swabs were collected from healthy children in Tehran between December 2011 and August a2012, and 100 clinical samples were collected. Identification was performed by biochemical and molecular tests. Serotyping was done by multiplex PCR. We designed primers based on the sequences available for the routine capsular types and combined them into six multiplex PCR.

**Results:**

From 150 nasopharyngeal swabs, 40 isolates of *Streptococcus pneumoniae* were identified after identification tests. Thirty six clinical isolates were also detected among clinical samples. Four serotypes (19A, 6, 3, 23F) of *S. pneumoniae* accounted for 55.7% of both sets of strains isolated from nasal carriage and clinical samples. Serotype 19A was the most common serotype among both groups.

**Conclusion:**

The multiplex PCR approach was successfully adapted to identify serotypes from more than 91% of the isolates tested. Among *S. pneumoniae* isolates in Tehran, the most prevalent serotypes were similar among carriage and invasive isolates. Continued monitoring of common serotypes of *Streptococcus pneumoniae* is essential for future vaccine formulation in Iran.

## INTRODUCTION

*Streptococcus pneumoniae* is an important infectious agent causing of life-threatening infection diseases worldwide, particularly in children and the elderly people (1). World Health Organization (WHO) has reported that nearly 1 million children die of pneumococcal disease annually, mostly in developing countries (2). Several diseases are caused by *S. pneumoniae* including sepsis, pneumonia, meningitis, otitis media, sinusitis and bronchitis (2, 3). Antimicrobial resistance in *S. pneumoniae* has increased over the last three decades (4). The antibiotic-resistant strains are often associated with multiple serotypes and the rates of antibiotic resistance varies geographically (4, 5). The increase of antibiotic resistance has complicated treatment of pneumococcal infections (6).

*S. pneumoniae* can produce at least 90 capsular serotypes (7), but only a few of these cause most cases of invasive diseases (8). Identification of these serotypes is essential for production of the new generation of conjugate vaccines and treatment protocols (5). There are geographical differences in prevalence and distribution of serotypes (9). Hence, it is necessary to determine the prevalent serotypes of *S. pneumoniae* in different geographical areas.

Pneumococcal polysaccharide vaccines and pneumococcal conjugate vaccines are two types of vaccines that are used in control of invasive diseases. Polysaccharide vaccines are not effective against otitis media or nasopharyngeal carriage and are poorly immunogenic in children less than age 2 years (10, 11). Pneumococcal conjugate vaccine 7-valent (PCV7) was routinely used in USA and Europe, but it has not been used since March 2010, and vaccination with PCV13 has started in many countries since 2010 (6). Use of an effective conjugate vaccine during infancy reduced the rates of invasive pneumococcal infections and antibiotic resistance associated with serotypes (12). However, infections caused by other serotypes have increased (4). Although previous studies showed that distribution of pneumococcal diseases and the rates of antimicrobial resistance in Iran are high (13, 14), a few studies have been performed on the serotypes distribution of *S.pneumoniae* in Iran.

Traditionally, the Quellung reaction is used for serotyping of *S. pneumoniae*. However, the high cost and require skilled technicians have stimulated the need for the development of PCR-based serotyping systems (15–17). Molecular methods such as multiplex PCR are faster and simpler as well as have higher sensitivity and lower cost than the cultural methods. Furthermore, Multiplex PCR can be used directly on clinical samples obtained from culture-negative patients (18). The aim of this study was to determine the prevalent pneumococcal serotypes and to compare serotypes in healthy carrier children and clinical samples of *S. pneumoniae* by Multiplex-PCR method.

## MATERIALS AND METHODS

### Bacterial isolates

In a cross-sectional study, 150 nasopharyngeal swabs were collected from healthy children less than 5 years of age having referred to 4 main care centers (Torkamani, Shobeir, Hazrate Roghayyeh, Ameneh) and Children's Medical Center of Tehran from December 2011 to August 2012. Nasopharyngeal swabs were kept in Stuart transport medium (Oxoid Limited, Hampshire, England) which were sent to the Department of Bacteriology in Pasteur Institute of Iran. Nasopharyngeal swabs were inoculated onto 5% sheep blood agar and incubated overnight at 37°C in 5% CO_2_. The colonies that were morphologically similar to *S. pneumoniae* were sub-cultured onto 5% sheep blood agar. One hundred clinical samples including blood, cerebrospinal and pleural fluids were collected from patients of Imam Khomeini, Sina, Shohada and Hazrate Rasoul hospitals during September 2008-March 2010. The clinical samples were obtained from invasive and non-invasive infection of both pediatric and adult patients.

### Phenotypic and Molecular Identification Methods

Identification of *S. pneumoniae* isolates were performed by biochemical methods such as Gram staining, colony morphology (alpha-hemolytic, small, gray, and showing mucoid colonies), optochin susceptibility, and bile solubility. Optochin disks (6 mm; MAST diagnostics, Bostle, Mersey side) were applied to 5% sheep blood agar. After overnight incubation at 37°C in 5% CO_2_ atmosphere, isolates displaying zones =14 mm in diameter were considered as *S. pneumoniae*. Complete clearing of heavy bacterial suspensions (1 ml) matching the 2.0 McFarland turbidity in the deoxycholate tube after incubation at room temperature for 15 min was indicative of *S. pneumoniae*. Molecular tests were used for confirmation of identification of isolates by PCR for *cpsA* gene as described previously (19). *S. pneumoniae* ATCC 6305 was used as control strain in all of identification tests.

### Preparation of chromosal DNA

Genomic DNAs were extracted using a DNA extraction Kit (Metabion™ international AG, Germany) according to the manufacture's protocol. Finally, the harvested DNA pellet was re-suspended in RNase- Tris-EDTA to provide 70 µl of DNA sample.

### Multiplex PCRs

Primers were used based on the sequences available for the capsular types 1, 3, 4, 5, 6AB, 7F, 9V, 10A, 11, 12, 14, 15, 16, 18C, 19F, 19A, 22, 23F, 33F, and 35B and combined them into six multiplex PCR according to previously outlined methods (20). These primers were grouped based on major serotypes reported among invasive and non-invasive pneumococci recovered in different geographical regions. To confirm the results of our multiplex PCR, we used reference standard strains for each reaction of multiplex PCR. These strains included ATCC 6305, ATCC 6301, ATCC 49619, ATCC 49136 and ATCC 700677. Multiplex PCR amplifications were performed in 25 µl reaction mixture containing 0.5 µl of dNTPs (10 mM), 0.5 µl of each primer (10 pmol), 2.5 µl PCR buffer (10x), 1.5 µl MgCl_2_ (25 mM), 0.2 µl Taq DNA polymerase (5U/µl) (Fermentas, Lithuania) (Tables 1 and 2). The PCR cycle was 94°C for 4 min followed by 30 amplification cycles of 94°C for 45 s, 56°C for 45 s, and 72°C for 2 min. PCR products were separated in a 1% agarose gel for 1 h at 100 V, stained with ethidium bromide and detected by UV transluminator.

**Table 1:** Classification of serotypes to molecular capsular typing, indicating the five reactions and the serotypes detected in each reaction.

Reaction 1				Reaction 2				Reaction 3				Reaction 4				Reaction 5			Reaction 6
**19A**	**3**	**6A/6B**	**22**	**4**	**14**	**12**	**9V**	**23F**	**11**	**33F**	**7F**	**19F**	**16**	**18C**	**35B**	**10A**	**1**	**15**	**5**

**Table 2:** Oligonucleotide primers used in this study.

Primer pair	Primer Sequence (5'–3')	References
**1**	F: CTC TAT AGA ATG GAG TAT ATA AAC TAT GGT TA R: CCA AAG AAA ATA CTA ACA TTA TCA CAA TAT TGG C	Pai et al. 2006
**3**	F: ATG GTG TGA TTT CTC CTA GAT TGG AAA GTA G R: CTT CTC CAA TTG CTT ACC AAG TGC AAT AAC G	Pai et al. 2006
**4**	F: CTG TTA CTT GTT CTG GAC TCT CGA TAA TTG G R: GCC CAC TCC TGT TAA AAT CCT ACC CGC ATT G	Jiang et al. 2001
**5**	F: ATA CCT ACA CAA CTT CTG ATT ATG CCT TTG TG R: GCT CGA TAA ACA TAA TCA ATA TTT GAA AAA GTA TG	GenBank: AY336008
**6A/B**	F: AAT TTG TAT TTT ATT CAT GCC TAT ATC TGG R: TTA GCG GAG ATA ATT TAA AAT GAT GAC TA	Jiang et al. 2001
**7F**	F: CCT ACG GGA GGA TAT AAA ATT ATT TTT GAG R: CAA ATA CAC CAC TAT AGG CTG TTG AGA CTA AC	Pai et al. 2006
**9V**	F: CTT CGT TAG TTA AAA TTC TAA ATT TTT CTA AG R: GTC CCA ATA CCA GTC CTT GCA ACA CAA G	Pai et al. 2006
**10**	F: GGT GTA GAT TTA CCA TTA GTG TCG GCA GAC R: GAA TTT CTT CTT TAA GAT TCG GAT ATT TCT C	Pai et al. 2006
**11**	F: GGA CAT GTT CAG GTG ATT TCC CAA TAT AGT G R: GAT TAT GAG TGT AAT TTA TTC CAA CTT CTC CC	Pai et al. 2006
**12**	F: GCA ACA AAC GGC GTG AAA GTA GTT G R: CAA GAT GAA TAT CAC TAC CAA TAA CAA AAC	Pai et al. 2006
**14**	F: CTT GGC GCA GGT GTC AGA ATT CCC TCT AC R: GCC AAA ATA CTG ACA AAG CTA GAA TAT AGC C	Pai et al. 2006
**15**	F: ATT AGT ACA GCT GCT GGA ATA TCT CTT C R: GAT CTA GTG AAC GTA CTA TTC CAA AC	Pai et al. 2006
**16**	F: CTG TTC AGA TAG GCC ATT TAC AGC TTT AAA TC R: CAT TCC TTT TGT ATA TAG TGC TAG TTC ATC C	Pai et al. 2006
**18C**	F: CTT AAT AGC TCT CAT TAT TCT TTT TTT AAG CC R: TTA TCT GTA AAC CAT ATC AGC ATC TGA AAC	Pai et al. 2006
**19F**	F: GTT AAG ATT GCT GAT CGA TTA ATT GAT ATC C R: GTA ATA TGT CTT TAG GGC GTT TAT GGC GAT AG	Guidolin et al. 1994
**19A**	F: GTT AGT CCT GTT TTA GAT TTA TTT GGT GAT GT R: GAG CAG TCA ATA AGA TGA GAC GAT AGT TAG	Morona et al. 1999
**22**	F: GAG TAT AGC CAG ATT ATG GCA GTT TTA TTG TC R: CTC CAG CAC TTG CGC TGG AAA CAA CAG ACA AC	Pai et al. 2006
**23F**	F: GTA ACA GTT GCT GTA GAG GGA ATT GGC TTT TC R: CAC AAC ACC TAA CAC ACG ATG GCT ATA TGA TTC	Ramirez et al. 1998
**33F**	F: GAA GGC AAT CAA TGT GAT TGT GTC GCG 181 338 R: CTT CAA AAT GAA GAT TAT AGT ACC CTT CTA C	Kong et al. 2003
**35B**	F: GAT AAG TCT GTT GTG GAG ACT TAA AAA GAA TG R: CTT TCC AGA TAA TTA CAG GTA TTC CTG AAG CAA G	Pai et al. 2006

## RESULTS

### Identification of *Streptococcus pneumoniae* isolates

**Fig. 1:**
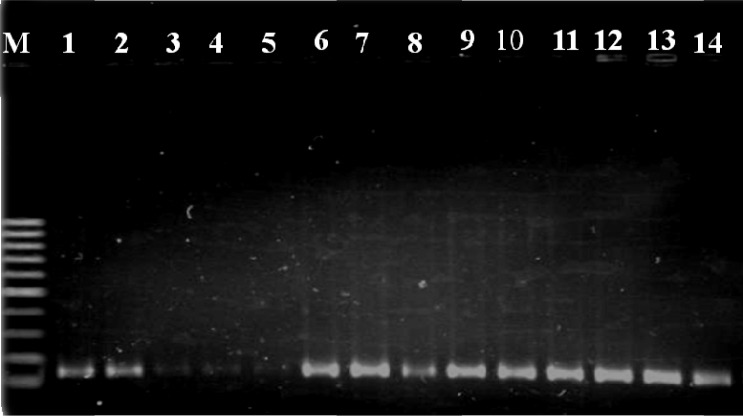
Molecular identification of *Streptococcus pneumoniae* isolates by detection of *cpsA* gene. Lane M, 100 bp ladder; Lane 1, Control strain (*S. pneumoniae* ATCC6305); Lanes 2 to 14, alpha-hemolytic and optochin susceptible isolates detected as *S.pneumoniae*.

From 150 nasopharyngeal swabs, 40 isolates were isolated and identified as *S. pneumoniae* using standard tests. In addition, 36 clinical isolates were identified among patients. All pneumococcal strains were optochin susceptible and bile soluble. Presence of *cpsA* gene was confirmed in all 76 *S. pneumoniae* isolates (Fig. 1). Overall prevalence of nasal carriage among children was 26.6%.

**Fig. 2:**
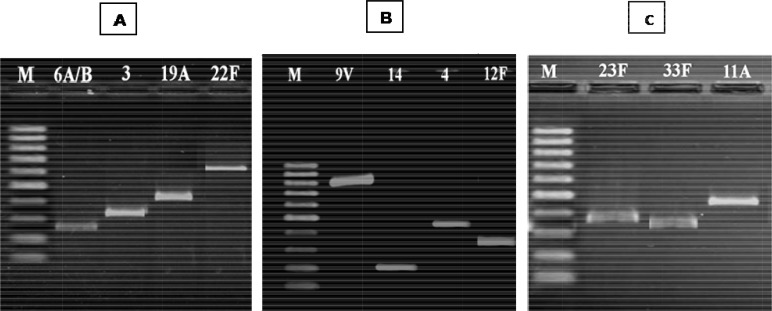
Multiplex PCR reactions 1, 2, and 3 for serotyping of *Streptococcus pneumoniae* isolates. A, Reaction 1; B, Reaction 2; C, Reaction 3. The specific serotypes identified in each reaction are indicated in above the lanes.

### Multiplex PCRs and Serotypes distribution

Of the 76 isolates of *S. pneumoniae*, 70 isolates (92.1%) were serotyped with the available specific primers by multiplex PCR (Fig. 2). Among *S. pneumoniae* strains isolated from nasal carriage, 37 (92.5%) were serotyped successfully by multiplex PCR. Similarly, multiplex PCR was successful in serotyping of 33 of 36 clinical isolates of *S. pneumoniae* (91.6%). All results were consistent with the scheme containing classification of serotypes in Table 1. Primers concentration in each multiplex PCR reaction was variable from 0.5 µM to 1.5 µM to obtain the best result.

Fifty one percent of strains (36 of 70) were serotyped in the first reaction of multiplex PCR. This rate for reactions 2, 3, 4, and 5 was 21.4%, 11.4%, 7.1%, and 4.2%, respectively. No strains were serotyped by reaction 6 that harbored specific primer for serotype 5.

Four serotypes (19A, 6, 3, 23F) accounted for 55.7% of both sets of strains isolated from nasal carriage and clinical specimens. Among *S. pneumoniae* isolated from nasal carriage, Serotype 19A was the most common serotype (17.5%), followed by serotypes 6 (15%) and 3 (15%). The most prevalent serotypes in clinical isolates of *S. pneumoniae* were 19A (22.2%), 6 (13.8%), and 23F (11.1%), respectively (Table 3). Serotypes 5, 7F, 10, 15, 16, 18C, and 35B were included in multiplex PCR, but were not identified among the isolates.

**Table 3:** Distribution of serotypes of *Streptococcus pneumoniae* strains.

Serotype	Pediatric carriage isolates (n = 40)		Clinical strains(n = 36)		Total (n = 76)	

	number	%	number	%	number	%
1	1	2.5	2	5.5	3	3.9
3	6	15	1	2.7	7	9.2
4	3	7.5	1	2.7	4	5.2
6A/B	6	15	5	13.8	11	14.4
9V	2	5	1	2.7	3	3.9
11A	1	2.5	2	5.5	3	3.9
12F	2	5	1	2.7	3	3.9
14	3	7.5	2	5.5	5	6.5
19A	7	17.5	8	22.2	15	19.7
19F	2	5	3	8.3	5	6.5
22F	1	2.5	2	5.5	3	3.9
23F	2	5	4	11.1	6	7.9
33F	1	2.5	1	2.7	2	2.6
Nontypeable	3	7.5	3	8.3	6	7.9

## DISCUSSION

*Streptococcus pneumoniae* is a common cause of respiratory infections requiring hospitalization in young children worldwide with increasing rates of antibiotic resistance (21). Generally, serotype determination of *S. pneumoniae* is performed by culture of the organism followed by serological detection of the capsular type by quellung test. This method of serotyping is not widely feasible due to the high cost of antisera, difficulties in interpretation, and requirement of technical expertise (20). The development of PCR-based serotyping has the potential to overcome some of the difficulties associated with the conventional serologic method.

In this survey, multiplex PCR was successful in serotyping of *S. pneumoniae* isolates. However, 6 isolates (7.8%) could not be typed by multiplex PCR test. These isolates could be non-serotypeable, but it is important to note that multiplex PCR system used in this study was designed to only detect 20 common serotypes which were previously reported (20, 22–24). As expected, the first three reactions in our multiplex PCR scheme detected the major previously reported serotypes from different countries. Interestingly, among the 76 isolated *S. pneumoniae* strains in this study, 83.8% were serotyped following three reactions of multiplex PCR indicating high sensitivity of this method in serotyping of *S. pneumoniae* strains. Similar to our findings, Pai et al (20) showed that these three reactions were able to detect more than 60% of the strains in the United States. Furthermore, using the first three reactions of this scheme in the Latin America, 89.1% of *S. pneumoniae* isolates were accurately serotyped (22). These results show that multiplex PCR can reduce the costs of serotyping compared to the conventional serotyping methods.

In this study, serotype distribution was examined in both carriers and clinical isolates. MP-PCR allowed serotyping in 37 (92.5%) of 40 nasopharyngeal carrier of *S. pneumoniae* in healthy children less than five years-old, and 33 (91.6%) of 36 with invasive pneumococcal disease (IPD). Both groups showed 13 serotypes of 20 serotypes examined. These serotypes were similar in both groups indicating the importance of nasopharyngeal colonization in the development of serious pneumococcal infections. However, there were differences in the position of some serotypes in the two groups that may be due to low number of specimens. To determine the actual position of serotypes, serotyping should be done on more specimens.

The serotype distribution in this study is not similar to that reported in previous study of Iran (14). The differences may be due to the use of different methods for serotyping. In the previous study, serotyping had been performed using antisera.

Among *S. pneumoniae* isolates in Tehran, Five serotypes were included about 60% of carriage and invasive isolates. Similarly, In Brazil, USA and UK a few pneumococcal serotypes has been related to the majority of nasopharyngeal carriage and invasive disease (24–26), In contrast, in Asia a wide variety of serotypes has been related to nasopharyngeal carriage and invasive disease (27, 28). In the present study, serotypes 19A and 6 were the most common serotypes in carriers and clinical isolates. High prevalence of serotype 3 was shown among carrier isolates, whereas it was rarely found in clinical isolates. Overall, the serotypes detected among the nasopharynx and serotypes causing invasive disease often are represented in the PCV13. Moreover, results showed that 48.5% of the pneumococcal serotypes were from serotypes of pneumococcal conjugate 7-valent vaccine (4, 6B, 9V, 14, 18C, 19F, 23F). However, Serotype 18C was not found among *S. pneumoniae* isolates.

Previous studies indicate that young children in Iran experience a high prevalence of *S. pneumoniae* carriage rate. A study of 1300 healthy children less than ten years-old in Tehran, Iran showed pneumococcal nasopharyngeal carriage rate of 44.1% (14). Another study showed a lower nasopharyngeal carriage rate (15.7%) for *S. pneumoniae* in Iranian healthy adolescents that decreased with age (13). Despite the high prevalence of carriage in Iranian children, so far vaccination has not been carried out in this country. Therefore, continued monitoring of common serotypes of *S. pneumoniae* is essential for future vaccine formulation in Iran. Since the serotypes distribution are similar among both nasopharyngeal carrier and clinical isolates in Tehran, epidemiological studies to investigate the prevalent serotypes in nasopharyngeal carriers could aid the development of a suitable vaccine for prevention of invasive pneumococcal diseases.
